# Adaptation and distribution of cardioplegia practices in Thailand during the COVID-19 pandemic: insights from a nationwide survey

**DOI:** 10.1051/ject/2025030

**Published:** 2025-12-17

**Authors:** Narongrit Kantathut, Parinya Leelayana, Piya Samankatiwat

**Affiliations:** 1 Division of Thoracic and Cardiovascular Surgery, Department of Surgery, Ramathibodi Hospital 270 Rama VI Road Ratchathewi Bangkok 10400 Thailand

**Keywords:** Cardioplegia, Myocardial protection, COVID-19, Cardiac surgery, Thailand, HTK, del Nido, St. Thomas cardioplegia

## Abstract

*Background*: Cardioplegia is essential for myocardial protection during cardiac surgery. The COVID-19 pandemic disrupted supply chains, affecting the availability of commercial cardioplegia solutions in Thailand and prompting institutions to modify their strategies. This study evaluates the distribution, selection, and adaptation of cardioplegia practices among Thai cardiac surgical centers during the pandemic. *Methods*: A nationwide survey was conducted in cardiac surgical centers performing ≥100 cases per year. Data on cardioplegia availability, usage, and preferences across different surgeries were collected via direct or telephone interviews with surgeons or perfusionists. Descriptive statistical analyses were applied. *Results*: St. Thomas-based cardioplegia remained the most widely used (95%), with 77.1% of institutions preparing custom formulations due to supply shortages. Histidine-tryptophan-ketoglutarate (HTK) was the second most used (76%), particularly in aortic and complex congenital surgeries, followed by del Nido cardioplegia (27%), often in modified formulations. Most centers (74%) used two to three cardioplegia solutions. Blood cardioplegia was preferred for coronary artery bypass grafting (89.2%) and valve procedures (78.4%), whereas HTK dominated in aortic (54.1%) and complex congenital surgeries (71.4%). *Conclusion*: Despite the pandemic, St. Thomas-based cardioplegia remained dominant in Thailand, with increasing reliance on HTK and modified del Nido cardioplegia. The widespread use of custom-made cardioplegia highlights the impact of supply chain disruptions. Post-pandemic studies are essential to evaluate long-term adaptations and refine myocardial protection strategies.

## Glossary of abbreviations

CABG: Coronary Artery Bypass Grafting
COVID-19: Coronavirus Disease 2019
HTK: Histidine-Tryptophan-Ketoglutarate
IQR: Interquartile Range
SD: Standard Deviation

## Introduction

Cardioplegia is a cornerstone of myocardial protection during cardiac surgery, enabling surgeons to perform complex procedures on a still and bloodless heart while preserving myocardial viability and function. The composition and delivery of cardioplegia solutions vary across regions and institutions, influenced by patient demographics, surgical practices, and resource availability. The COVID-19 pandemic significantly disrupted healthcare systems worldwide, affecting the supply chain and standard practices in cardiac surgery [1]. These disruptions necessitated adaptations in the selection and distribution of cardioplegia solutions to mitigate resource limitations while ensuring optimal patient outcomes.

The pandemic posed unique challenges to Thailand’s healthcare infrastructure, with hospitals facing shortages of essential components such as Plasma-Lyte A (Baxter Healthcare Corporation, Deerfield, IL) and other imported additives commonly used in cardioplegia formulations [2–6]. Additionally, widely used cardioplegia solutions, including DBL^TM^ Sterile Cardioplegia Concentrate (Hameln Pharmaceuticals GmbH, Hameln, Germany) or St. Thomas cardioplegia, became unavailable due to logistical constraints, as distributors canceled imports during the pandemic. In response, many cardiac centers transitioned to alternative solutions, such as del Nido cardioplegia and Histidine-tryptophan-ketoglutarate (HTK) solution (Custodiol^®^; Koehler Chemi, Alsbach-Haenlien, Germany), which provided both logistical and clinical advantages in resource-constrained environments [5, 7–10]. These adaptations highlight broader trends in myocardial protection, emphasizing the importance of flexibility and innovation in cardioplegia protocols.

To examine these adaptations, this study utilized data from a nationwide survey of cardiac centers in Thailand. The survey assessed the distribution, utilization, and decision-making processes regarding cardioplegia practices during the COVID-19 pandemic. By analyzing how healthcare institutions modified their approaches in response to supply chain disruptions and clinical demands, this study aims to provide valuable insights into lessons learned and their implications for future resource management and protocol development in cardiac surgery.

## Materials and methods

### Ethical statement and inclusion criteria

Ethical approval was obtained from the institutional review board of Ramathibodi Hospital, Mahidol University (reference ID: COA. MURA2021/279) prior to the commencement of the study. Surveys were conducted across cardiac surgical centers in Thailand that performed more than 100 cases per year to ensure the inclusion of centers with significant surgical volumes and to provide robust and representative insights. The survey methodology involved direct or telephone interviews with surgeons or perfusionists working in these centers. As this study involved a survey of institutional practices without patient data collection, informed consent was not required. All participating institutions remained anonymous, and the study complied with all ethical guidelines.

### Survey population and response rate

According to the 2021 registry of the Society of Thoracic Surgery of Thailand, a total of 99 cardiac surgical centers were operating nationwide. Of these, 37 centers reported performing more than 100 cardiac surgical cases annually and were therefore eligible for inclusion in this study. Surveys were conducted between May 2021 and March 2022. All 37 eligible centers were directly contacted, and responses were obtained from each, resulting in a 100% response rate within this cohort. To ensure accuracy and representativeness, the survey was initially directed to the chief perfusionist or cardiac surgeon at each center. For questions concerning specialized procedures, such as aortic or congenital surgeries, respondents were encouraged to consult with or forward the questions to the appropriate subspecialist, such as the head of the respective surgical division. This strategy enhanced the reliability of the data by ensuring that the responses accurately reflected institutional practices across the spectrum of cardiac procedures.

### Questionnaire

The questionnaire assessed the following key aspects:

1. Availability of cardioplegia solutions – Evaluating the accessibility and supply of different cardioplegia formulations across cardiac surgical centers.
2. Number of cardioplegia solutions used in each institution – Identifying the variety and quantity of cardioplegia solutions utilized.
3. Number of custom-made cardioplegia solutions available – Assessing the prevalence and usage of institutionally prepared cardioplegia formulations.
4. Preferred cardioplegia selection – Identifying the cardioplegia solutions used for various types of cardiac surgeries, including coronary artery bypass grafting (CABG), valve procedures, aortic surgery, simple congenital heart surgery, and complex congenital heart surgery. Each institution could have more than one preferred cardioplegia solution for each type of operation.

Simple congenital heart surgeries involve defects that can be corrected with straightforward procedures, typically requiring a single operation with low to moderate surgical risk and minimal alterations to cardiac anatomy and function. These procedures have a high likelihood of complete correction. Examples include atrial septal defect closure, ventricular septal defect closure, patent ductus arteriosus ligation, coarctation of the aorta repair, and pulmonary valve stenosis repair.

In contrast, complex congenital heart surgeries involve intricate procedures due to the severity of the defect, often requiring extensive reconstruction, multiple-stage surgeries, or long-term management. These surgeries typically address abnormalities in multiple cardiac structures, significant alterations in circulation, or the need for artificial conduits, valves, or shunts. Examples of complex congenital heart surgeries include Tetralogy of Fallot repair, transposition of the great arteries correction (e.g., arterial switch operation), hypoplastic left heart syndrome staged palliation (Norwood, Glenn, and Fontan procedures), total anomalous pulmonary venous return repair, double outlet right ventricle correction, atrioventricular septal defect repair, and truncus arteriosus repair.

## Statistical analyses

Continuous data were presented using measures of central tendency such as mean (standard deviation [SD]) or median (interquartile range [IQR]). Categorical variables were represented as frequencies (%). The statistical analyses were conducted utilizing STATA version 14 (StataCorp, College Station, TX).

## Results

**Figure 1:**
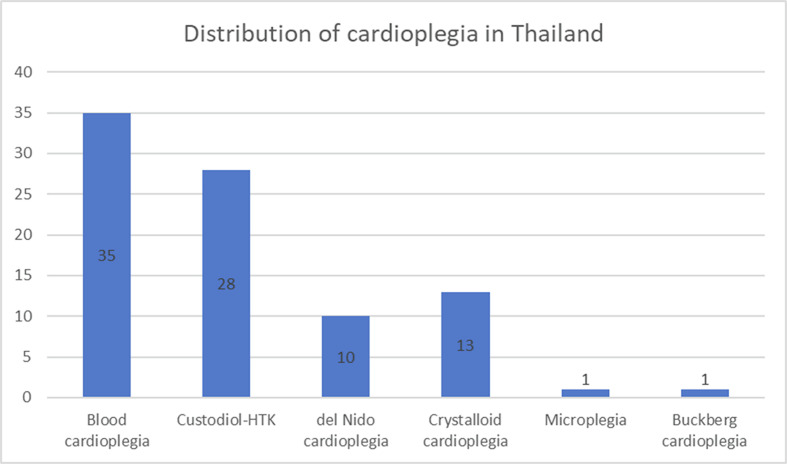
Availability of cardioplegia in Thailand (Blood cardioplegia is defined as the administration of St. Thomas-based cardioplegia, including DBL™ or custom-made formulations, mixed with blood prior to delivery. In contrast, crystalloid cardioplegia refers to the administration of St. Thomas-based cardioplegia delivered in its pure crystalloid form without the addition of blood).

**Figure 2:**
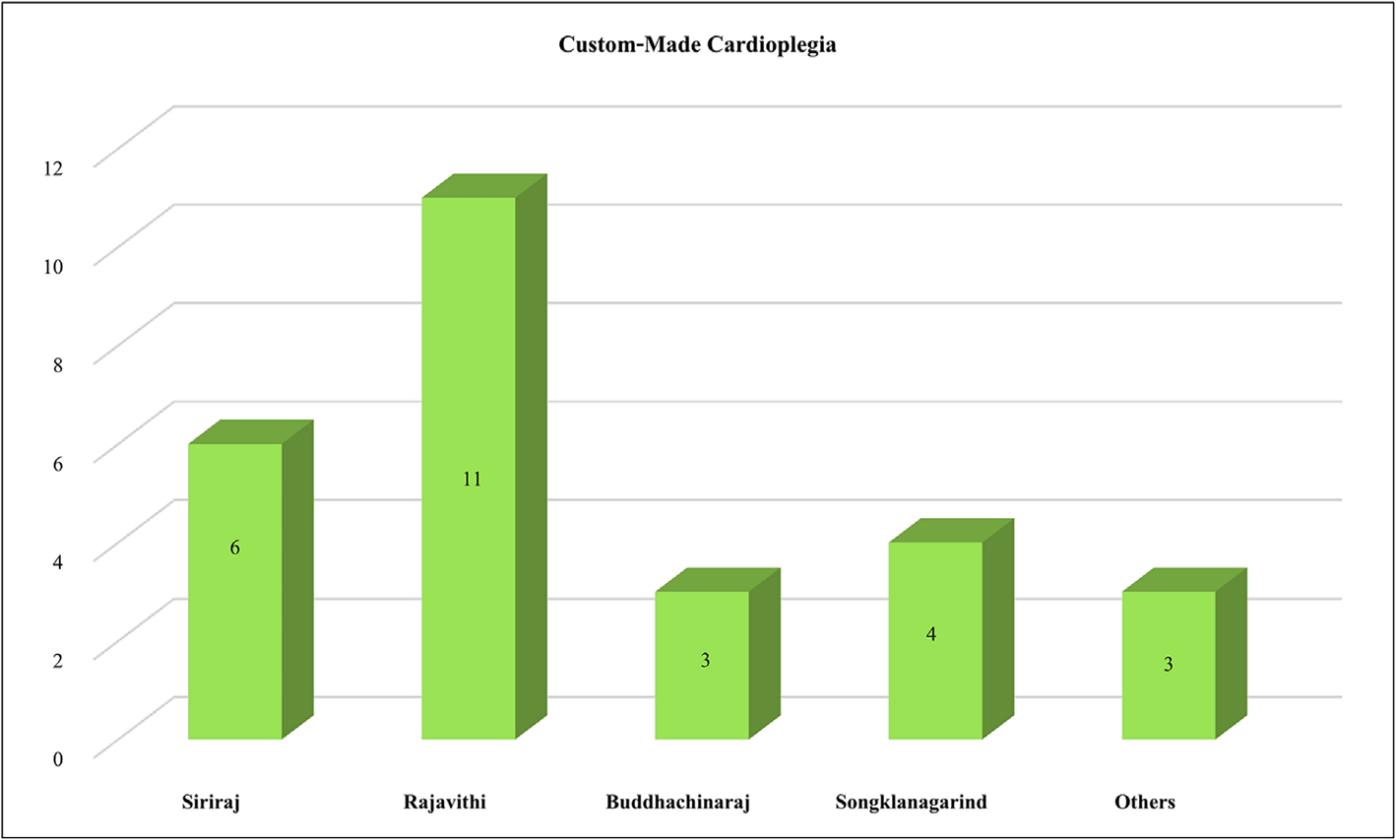
Custom-made formulations of St. Thomas-based cardioplegia. The names under each block represent the hospitals or cardiac surgical centers responsible for preparing and administering each formulation.

St. Thomas-based cardioplegia remains the most widely used cardioplegia solution in Thailand, with 95% of surveyed cardiac surgical centers reporting its use (Figure 1). Among these, 27 out of 35 (77.14%) institutions utilized custom-made formulations (Figure 2). HTK was identified as the second most frequently used cardioplegia solution, following St. Thomas-based formulations. Del Nido cardioplegia has demonstrated increasing utilization, emerging as the third most commonly used formulation.

**Figure 3:**
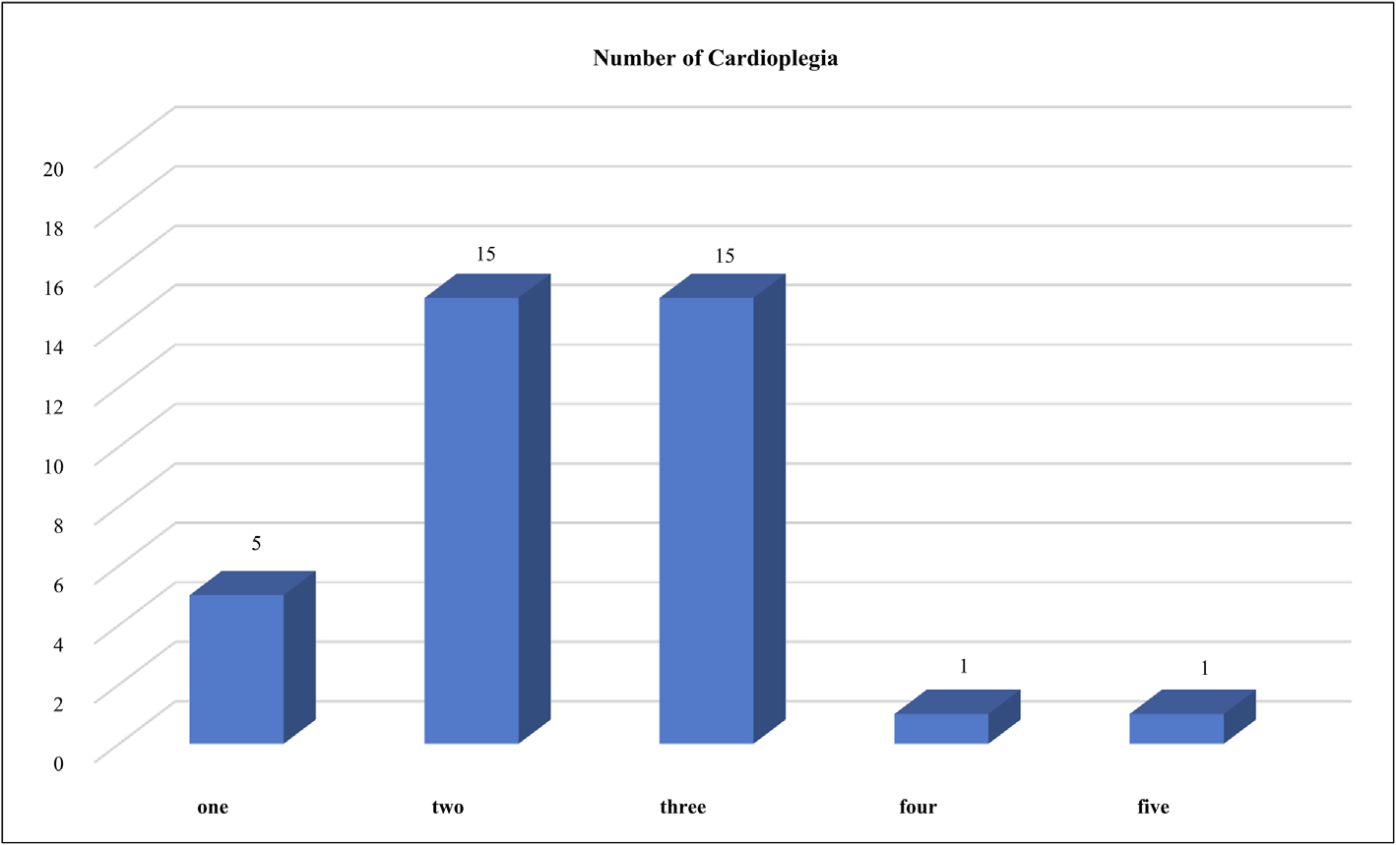
Number of cardioplegia solutions used per institution.

The majority of institutions reported using two to three different cardioplegia solutions in their practice. However, only one institution reported utilizing five different cardioplegia solutions (Figure 3).

**Figure 4:**
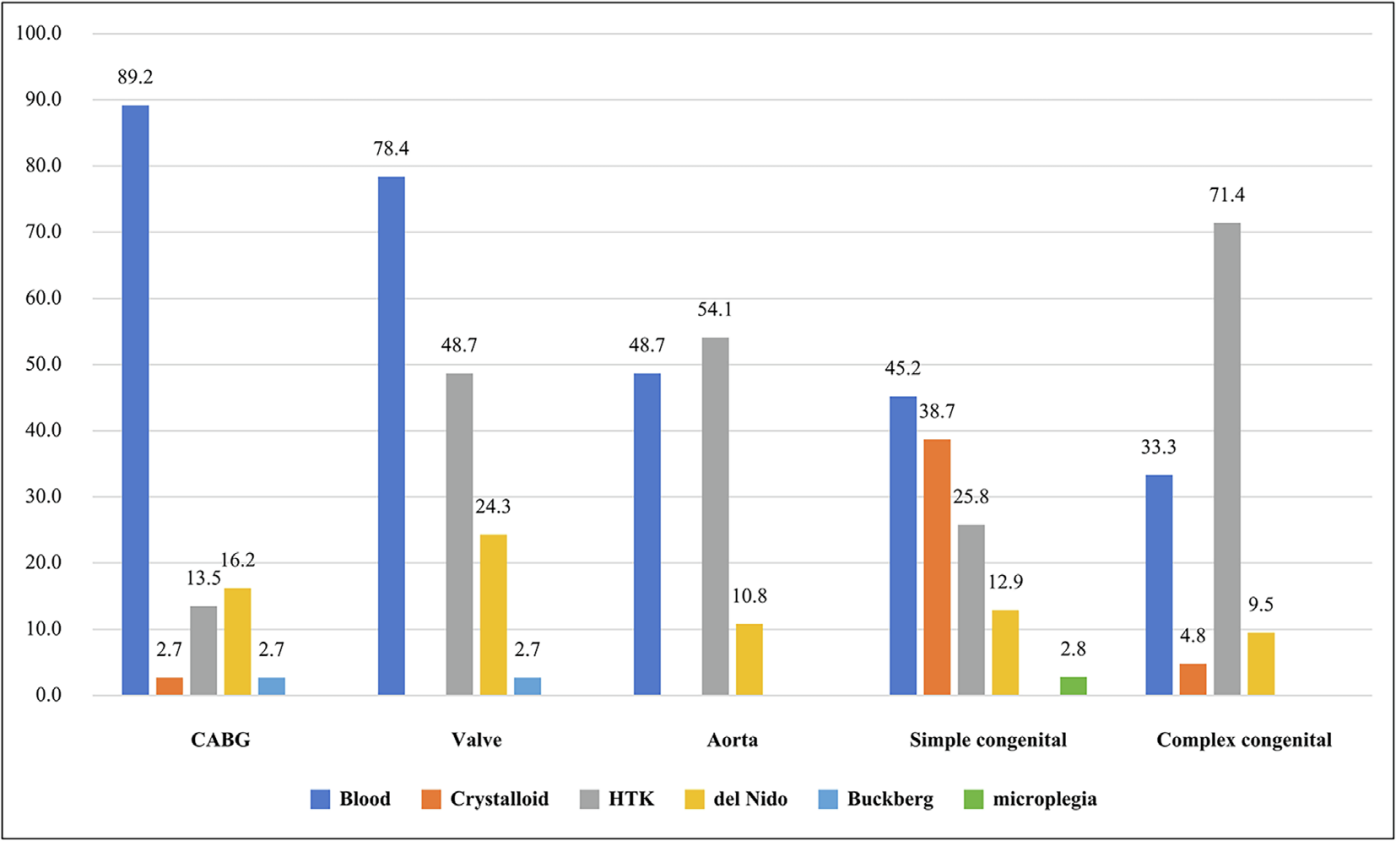
Preferred cardioplegia by procedure type. (CABG = Coronary artery bypass grafting, HTK = Histidine-Tryptophan-Ketoglutarate solution (Custodiol^®^))*.*

The distribution of preferred cardioplegia solutions for various types of cardiac surgeries is summarized in Figure 4. St. Thomas-based blood cardioplegia was the predominant choice for CABG (89.2%) and valve procedures (78.4%). HTK was the most utilized solution for aortic (54.1%) and complex congenital surgeries (71.4%). For simple congenital surgeries, there was a more even distribution, with St. Thomas-based blood cardioplegia (45.2%) and St. Thomas-based crystalloid cardioplegia (38.7%) is the primary choices. Del Nido cardioplegia was increasingly used across different procedures (9.5–24.3%).

## Discussion

This study provides a comprehensive overview of the distribution and selection of cardioplegia solutions used in Thailand during the COVID-19 pandemic. The findings reveal distinct trends in cardioplegia preferences across different surgical procedures and institutions, reflecting both historical practices and adaptations to logistical constraints.

### Prevalence of St. Thomas-based cardioplegia and custom-made solutions

St. Thomas-based cardioplegia remains the predominant choice among cardiac surgical centers in Thailand, with 95% of institutions reporting its use. This preference aligns with its long-established efficacy, cost-effectiveness, and widespread availability [3, 4, 8, 10]. However, a notable trend was the significant reliance on custom-made formulations, with 27 out of 35 institutions (77.14%) preparing their own variations of St. Thomas-based cardioplegia. This shift is largely attributed to the temporary unavailability of commercially prepared solutions, such as DBL^TM^ and other standard formulations, due to importation restrictions during the pandemic. The increased utilization of custom-made solutions highlights the adaptability of surgical centers in ensuring continuity of care despite supply chain disruptions.

### Adoption of alternative cardioplegia solutions

HTK emerged as the second most commonly used cardioplegia solution, reported in 76% of institutions. Its predominant application in aortic and complex congenital surgeries can be explained by its extended myocardial protection and single-dose administration, which minimizes interruptions during prolonged procedures [11]. Del Nido cardioplegia was reported in 27% of institutions, reflecting its increasing adoption in pediatric and congenital heart surgeries due to its simplified dosing regimen and myocardial protective benefits. However, it is important to note that the del Nido cardioplegia used in Thailand is not in its original formulation. Due to the unavailability of Plasma-Lyte, institutions have to modify its composition, which may affect its performance and clinical outcomes [2–6]. This modification raises important considerations regarding the standardization and efficacy of altered cardioplegia formulations.

### Institutional variability in cardioplegia selection

The survey revealed that most institutions employed a combination of two to three different cardioplegia solutions, allowing for tailored myocardial protection strategies based on the surgical procedure and patient profile. Only one institution reported using five different cardioplegia solutions, suggesting a highly diversified approach to myocardial protection. This variation in cardioplegia selection underscores the influence of institutional protocols, surgeon preferences, and resource availability. The increased adoption of multiple cardioplegia solutions within a single institution may indicate efforts to optimize surgical outcomes based on evolving evidence and surgical complexity.

### Preferred cardioplegia by procedure type

The selection of cardioplegia varied according to the type of cardiac surgery performed. St. Thomas-based blood cardioplegia was the dominant choice for CABG (89.2%) and valve procedures (78.4%). This preference aligns with previous studies demonstrating the benefits of blood-based cardioplegia, including superior oxygen delivery, buffering capacity, and reduced myocardial edema, particularly in ischemic hearts [12].

Conversely, HTK was the most commonly used cardioplegia for aortic surgery (54.1%) and complex congenital procedures (71.4%). These findings are consistent with its extended myocardial protective effects, which are particularly beneficial in surgeries requiring prolonged ischemic time.

For simple congenital heart surgeries, there was a more even distribution of cardioplegia choices, with 45.2% of institutions using St. Thomas-based blood cardioplegia and 38.7% using St. Thomas-based crystalloid cardioplegia. This variability suggests that institutions employ different cardioplegia strategies based on patient-specific factors, institutional experience, and available resources. Notably, the adoption of del Nido cardioplegia across multiple surgical categories, including adult procedures, reflects its expanding role beyond pediatric cardiac surgery [13–15].

### Impact of custom-made cardioplegia solutions

The study identified several institutions preparing their own custom-made cardioplegia formulations, likely as a response to supply chain disruptions during the pandemic. This trend was particularly prominent with St. Thomas-based solutions, where institutions adapted by compounding their own mixtures to maintain continuity in surgical care. While custom-made formulations provide flexibility and ensure the availability of essential resources, they also introduce variability in composition, which may impact clinical outcomes. The modification of del Nido cardioplegia, due to the absence of Plasma-Lyte, further underscores the challenges associated with maintaining standardized formulations in constrained supply environments. Future research should evaluate the efficacy and safety of these modified formulations to establish best practices for myocardial protection.

### Limitations

This study is limited to high-volume cardiac surgical centers performing more than 100 cases per year, potentially excluding smaller centers that may have distinct practices, resource constraints, or alternative cardioplegia strategies. Additionally, the survey-based methodology relies on self-reported data from participating institutions, which may introduce recall bias and variability in reporting practices. These factors may affect the generalizability of the findings across all cardiac surgical centers in Thailand.

Future research should aim to incorporate a broader and more diverse dataset, including lower-volume centers, to provide a more comprehensive assessment of cardioplegia practices. Furthermore, post-pandemic surveys will be essential to assess adaptations and the emergence of new cardioplegia strategies in response to supply chain disruptions and evolving surgical needs. Longitudinal studies evaluating long-term trends and clinical outcomes associated with different cardioplegia strategies are necessary to refine myocardial protection protocols and optimize patient care in cardiac surgery.

## Conclusion

This study highlights the predominant use of St. Thomas-based cardioplegia in Thailand, with a growing reliance on alternative solutions such as HTK and del Nido cardioplegia. The variation in cardioplegia selection across institutions underscores the need for standardized protocols while maintaining flexibility for case-specific myocardial protection strategies. The impact of supply chain disruptions during the COVID-19 pandemic further emphasizes the importance of contingency planning to ensure the consistent availability of essential surgical resources. Moving forward, post-pandemic surveys will be crucial in evaluating adaptations and the evolution of new cardioplegia strategies.

## Data Availability

The data underlying this article will be shared on reasonable request to the corresponding author.
